# Occupational heat exposure incidence and sociodemographic disparities for outdoor workers in the United States, 2010–2019

**DOI:** 10.1038/s41370-025-00813-y

**Published:** 2025-10-09

**Authors:** Abas Shkembi, Devon Payne-Sturges, Sung Kyun Park, Richard L. Neitzel

**Affiliations:** 1 Department of Environmental Health Sciences, University of Michigan School of Public Health, Ann Arbor, MI USA; 2 Department of Epidemiology, University of Michigan School of Public Health, Ann Arbor, MI USA

**Keywords:** Environmental Justice, Workplace Exposures, Climate Change, Geospatial Analyses, Non-chemical Stressors

## Abstract

**Background:**

Outdoor workers are at high risk of hazardous heat exposures and associated adverse health outcomes. Questions remain as to how many workers are exposed.

**Objectives:**

To estimate the yearly incidence of outdoor workers in the United States (US) exposed to hazardous heat from 2010 to 2019 and assess whether marginalized populations of workers are disproportionately exposed.

**Methods:**

We simulated the census tract-level, daily proportion of workers exposed to hazardous heat using multiple datasets containing (i) employment counts, (ii) work characteristics that influence susceptibility to heat exposure (e.g., metabolic rate), and (iii) county-level data on daily-average wet bulb globe temperature. Occupations were classified according to the US SOC structure. Overexposure was determined using ACGIH screening criteria for unacclimatized workers. Daily exposure proportions were summarized as annual, average exposure rates per 100 workers. At the census tract level, annual exposure rates and sociodemographic estimates were merged together to ecologically assess exposure disparities using generalized additive models. Estimates are publicly available alongside an interactive map (https://sph-umich.shinyapps.io/work-heat-2010-2019/).

**Results:**

Estimated daily incidence ranged from 0 to 7.3% of workers. Nationwide, there were an estimated 1.46 daily exposures per 100 workers from 2010 to 2019, equivalent to 5.44 billion worker-days. Nationwide exposure rates did not vary substantially year-to-year. Construction and Extraction (9.77 per 100 workers), Installation, Maintenance, and Repair (8.05 per 100 workers), and Farming, Fishing, and Forestry occupations (7.51 per 100 workers) had the highest estimated exposure rates. Regionally, estimated rates were highest in the South (3.26 per 100 workers), particularly in Florida and Texas. Low-income individuals, individuals without a high school diploma, foreign-born populations, and racial and ethnic minority individuals (particularly Hispanic) were disproportionately exposed.

**Significance:**

The frequency of occupational heat exposure, its disproportionate burden on marginalized workers, and the increasing impact of climate change suggests a critical and urgent need for occupational regulation and surveillance of heat exposure.

**Impact:**

- This study characterizes potentially hazardous heat exposures among outdoor workers for every census tract in the contiguous United States during 2010-2019. Current epidemiological and health impact studies of heat-related disease and mortality do not typically consider how occupational heat exposure may exacerbate adverse heat-related health outcomes in communities experiencing high heat. Our approach could reduce exposure misclassification bias in these studies. Policy-wise, these spatiotemporal estimates can support regulatory enforcement and consultation of occupational heat exposure by better targeting particular communities with high heat exposure. These estimates can also inform surveillance efforts and healthcare resources of state and local health departments.

## Introduction

Outdoor workers are among the most vulnerable to the effects of heat [1], typically due to the need to work during peak sun hours and a lack of access to air conditioning—the most effective way to control heat exposure [2, 3]. In 2022, approximately 45 million workers had regular exposure to the outdoors in the United States (US), accounting for roughly a third of counted workers [4]. Further, outdoor work is common across many sectors, such as construction, agriculture, mining, and transportation, meaning occupational heat affects multiple industries in the US [5]. The burden of heat on outdoor workers in the US is expected to rise by 2050 due to climate change, with an at least a three-fold, nationwide increase in the annual number of days with high heat exposure [6]. As a consequence, heat-related illnesses (e.g., heat stroke, heat exhaustion) and traumatic injuries and fatalities (often due to cognitive impairment from dizziness and fatigue) for outdoor workers are an increasingly common issue. Some evidence also suggests occupational heat stress can lead to chronic health impacts such as kidney disease [1, 7, 8]. These negative effects have led to the US Occupational Safety and Health Administration, the agency in the US government that regulates workplaces, to propose a nationwide heat injury and illness prevention standard [9]. As of 2024, the nationwide standard has not been promulgated, although six US states (California, Oregon, Washington, Colorado, Minnesota, and Maryland) have passed their own state-specific heat standards.

**Fig. 1:**
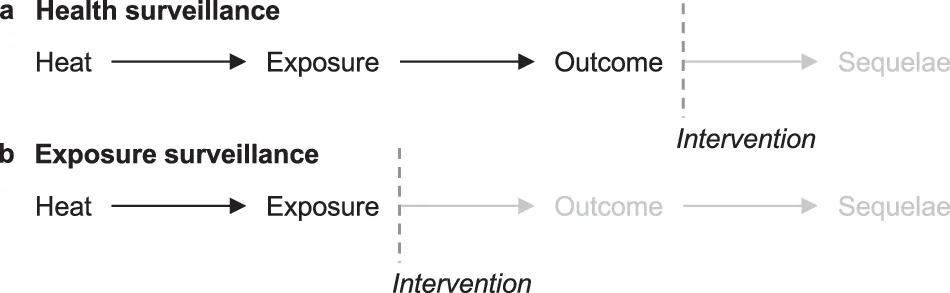
Comparison of public health prevention strategies for heat exposure. Conceptual model of heat-related (**a**) health surveillance vs (**b**) exposure surveillance and associated interventions.

Despite these adverse impacts on worker health and increased regulatory emphasis, it is unknown how many workers are currently exposed to hazardous levels of heat, as well as which workers may be disproportionately burdened. Current surveillance efforts have instead focused on monitoring heat-related injuries, illnesses, and deaths after heat exposure has occurred. While health surveillance can reduce the negative health and socioeconomic impacts of disease or injury, it prioritizes prevention efforts after adverse health outcomes have manifested (Fig. 1a). Heat-related health surveillance is also likely underreported [10]. There are multiple reasons for underreporting, such as (i) physicians, medical examiners, and coroners may not recognize heat as a cause of death [11], (ii) heat may impair cognitive ability that could indirectly increase the risk of other work-related injuries (e.g., falls) which may not be obviously linked to the heat exposure [12], (iii) workers may be disincentivized to report heat-related events, particularly those who are socioeconomically vulnerable (e.g., undocumented immigrants or low-wage workers) [13], (iv) workers may not be aware they can report heat-related incidents, and (iv) the effects of heat-related illness may be felt after leaving work, leading to the worker not associating the illness with their work shift. Without understanding the full magnitude of who is exposed, there will continue to be a lack of understanding of the true health burden of occupational heat exposure. Given these limitations of health surveillance, characterizing exposures can better inform approaches to reduce heat exposure, identify populations that are disproportionately exposed, and prevent adverse heat-related outcomes from occurring in the first place (Fig. 1b).

In this study, we characterized likely exposures to, and trends in, potentially hazardous heat workplace conditions from 2010 to 2019 for all outdoor workers in the contiguous United States (not including Alaska and Hawaii). Our approach provided not only national-level estimates, but also as granular as the county- and census tract-level. We used these census tract-level estimates to assess whether marginalized populations of workers (i.e., low-income, low educational attainment, racial and ethnic minority, foreign-born) were disproportionately exposed to occupational heat compared to non-marginalized populations.

## Methods

### Overview

We applied a Monte Carlo simulation approach to characterize the likely, daily exposure incidence to hazardous occupational heat from 2010 to 2019 for outdoor workers across all census tracts in the contiguous US. To achieve this, we integrated information on (i) the types of jobs people do and where, (ii) the typical work characteristics of these jobs that influence susceptibility to heat exposure, (iii) the typical heat exposures where people are working and (iv) whether these workers meet the American Conference of Governmental Industrial Hygienists (ACGIH) criteria for likely hazardous heat exposure.

**Fig. 2:**
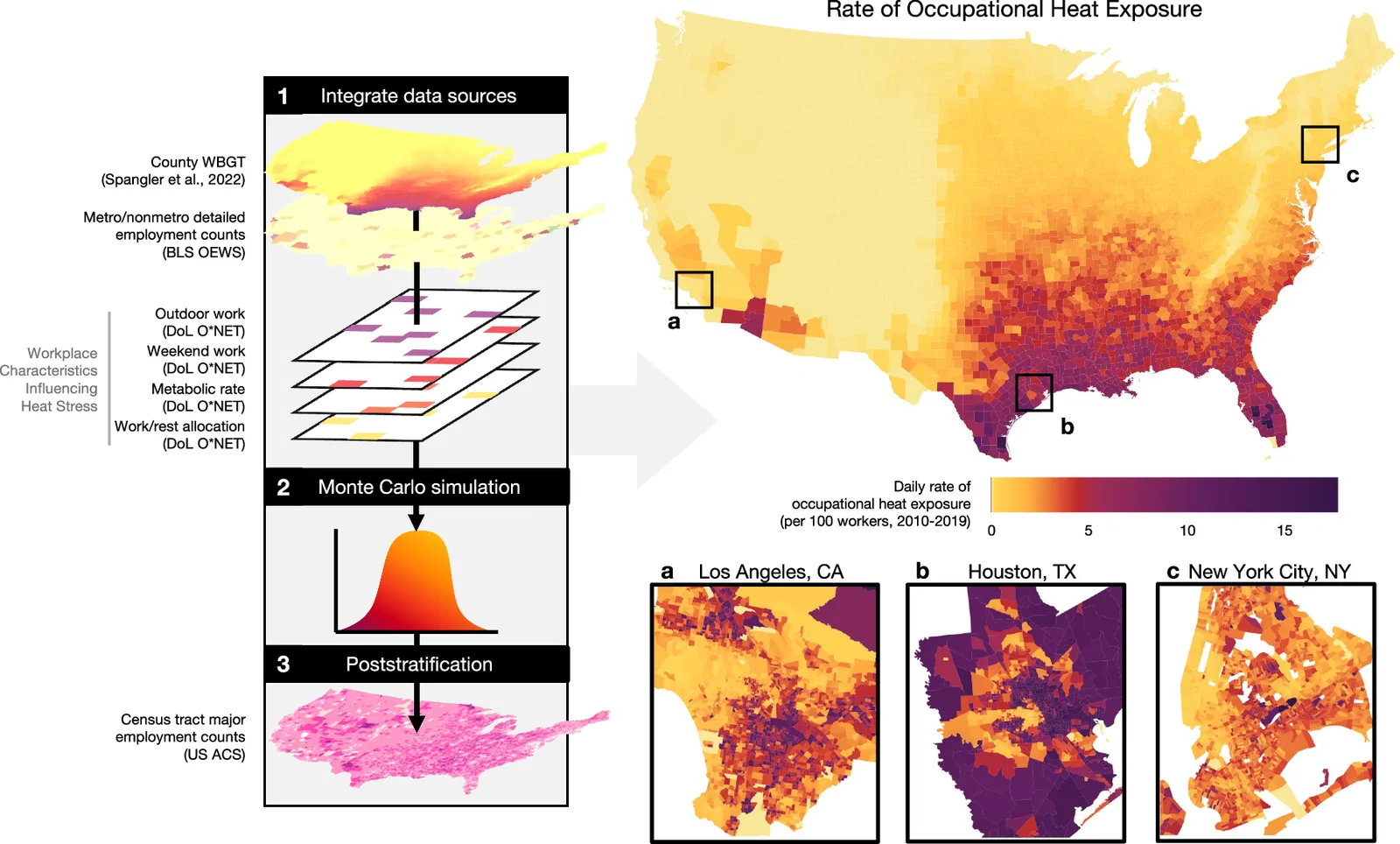
Data integration to estimate annual average rate of occupational heat exposure across the US. Example of census tract-level estimates are displayed for three large US cities: (**a**) Los Angeles, California; (**b**) Houston, Texas; and (**c**) New York City, New York. Note: WBGT wet bulb globe temperature, BLS OEWS Bureau of Labor Statistics Occupational Employment and Wage Statistics, DoL O*NET Department of Labor Occupational Information Network. US ACS United States American Community Survey.

We leveraged (i) employment counts by major occupational groups from the US American Community Survey, (ii) work characteristics from the Occupational Information Network (O*NET), and (iii) county-level data on daily-average wet bulb globe temperature (WBGT) for the contiguous US only, respectively. This approach estimated daily exposure incidence for every census tract in the contiguous US, which we aggregated both spatially (i.e., county, region, nationwide) and temporally (i.e., monthly, yearly). We characterized exposure estimates at the census tract level, rather than county, because our goal was to construct the most spatially granular estimates that are possible with publicly available data sources. A visual overview of this approach is displayed in Fig. 2.

### Data Integration

Occupations are commonly classified in the US following the US Standard Occupational Classification (SOC) structure. At the most granular level in 2010, non-military/civilian occupations are classified in 820 detailed SOC codes (e.g., security guards), which are grouped in 22 major SOC codes (e.g., protective services). Data in this study were integrated from multiple, disparate sources, each with varying levels of spatial, temporal, and occupational resolution. To our knowledge, the most spatially granular employment count data at the detailed SOC code level is the US Bureau of Labor Statistics (BLS) Occupational Wage and Employment Statistics (OEWS). This data source provided 2019 metropolitan/nonmetropolitan area employment counts of detailed Standard Occupational Classification (SOC) codes [14]. To link this data source with the county-level WBGT data, we assumed that the employment counts by detailed SOC codes were the same for the counties within a metro/nonmetro area. Overall employment counts were not altered at the county level because our main interest in this data source is the relative occupational composition of detailed SOC groups in a county. Assuming detailed SOC employment counts did not vary day-to-day and year-to-year from 2010 to 2019, we assigned each county-level, detailed SOC employment count a county-level, daily, mean WBGT estimate using previously published estimates (Supplementary Fig. B1) [15], which were available for the contiguous US only. We made this assumption because OEWS estimates are aggregated over time and are not intended to be assessed temporally.

We then assigned heat-related work characteristics to each detailed SOC. These were outdoor work, long/irregular work schedules, metabolic rate, and work/rest allocation, all derived from the 2019 O*NET (version 24.1) [16]. While indoor workers can also be at increased risk of heat stress, we only considered environmental heat exposure among outdoor workers in this analysis. Identifying those who could work long or irregular work schedules helped us identify potential occupations that might work on weekends, which is important to not bias our exposure estimates low by only considering weekdays. Metabolic rate and work/rest allocation were included because these two characteristics influence the WBGT threshold that places workers at risk of heat stress. Clothing is another important factor. For example, double-layered woven clothing may increase a worker’s internal temperature, while loose clothing likely will not. We did not consider clothing adjustment values because this information is not available in the O*NET. More details are provided in Appendix A of the Supplementary Materials regarding how each characteristic was derived. Long/irregular work schedules, metabolic rate, and work/rest allocation were each independent characteristics applied to a detailed SOC group; outdoor work was a probabilistic factor (i.e., the proportion of days in a year that a detailed SOC group works outdoors, p). Thus, for each day, we conducted Monte Carlo simulation with 200 iterations to assign a binary outcome of whether the outdoor workers may have been working outdoors on that day (i.e., ${Outdoor\; work} \sim {Binomial}(200,p)$). We chose 200 iterations because increasing iterations did not substantially improve estimation convergence (Appendix A, Figure A3).

If the simulation indicated that a detailed SOC group within a given county was working outdoors for a particular day, we then determined if the workers could be potentially exposed to hazardous heat using the ACGIH screening criteria for unacclimatized workers [17], which was determined as a function of a detailed SOC group’s metabolic rate, work/rest allocation, and the daily mean WBGT level (specific thresholds are displayed in Appendix A, Table A1). This assumes that all workers are unacclimatized to heat, which overestimates the number of individuals actually at risk of heat strain. However, the number of workers truly acclimatized to heat is unknown, so assuming all workers are unacclimatized provides a more realistic estimation of those potentially exposed to hazardous heat and is more inclusive of workers at increase susceptibility of heat strain. As a sensitivity analysis, we also re-ran our simulations assuming all workers are acclimatized (see Appendix B, Figs. B6, B8).

Thus, for each iteration-day, we estimated the number of workers exposed to hazardous levels of heat by detailed SOC group for each county in the contiguous US. The central estimate was the median across all 200 iterations. The unlikely, best-case scenario was the 2.5th percentile, while the unlikely, worst-case scenario was the 97.5^th^ percentile. We aggregated these detailed SOC estimated counts of workers exposed to the major SOC level. For each major SOC group, we thus estimated the proportion exposed (i.e., number of workers exposed divided by total number of workers) every county-day. Since our primary goal was to estimate occupational heat exposure at the most spatially granular level possible (i.e., census tract), we applied these daily-county-major SOC proportions to the census tract level using annual employment counts by major SOC group for each census tract using poststratification. Thus, for each day from 2010–2019, we estimated the proportion of workers potentially exposed to hazardous levels of occupational heat for every census tract in the contiguous US.

### Other covariates

For each year from 2010 to 2019, we extracted census tract-level estimates from 5-year, US American Community Survey data of the percentage of (i) racial and ethnic minorities (i.e., individuals who do not identify as non-Hispanic White); (ii) low-income individuals (i.e., <200% of the federal poverty limit); (iii) individuals with low educational attainment (i.e., without a high school diploma), and foreign-born populations. We further broke down the percentage of racial and ethnic minorities to four separate groups: non-Hispanic Black, non-Hispanic Asian, Hispanic, and all other minority race and ethnicity groups. To control for the likely non-working population of a census tract in our regression modeling, we extracted census tract-level estimates of the percentage of (i) individuals <16 years old, (ii) individuals >64 years old, and (iii) individuals who are unemployed, in the armed forces, or not in the labor force.

### Statistical analysis

#### Descriptive statistics

All analyses were conducted in R version 4.2.1. We summarized and visualized our estimations in a variety of ways. Treating each worker-day of exposure as an incident event, we aggregated the daily exposure proportions to monthly and yearly exposure rates per 100 workers.

$${Rate}=\frac{{\sum }_{i}{\sum }_{j}{{Exposures}}_{{ij}}}{{\sum }_{j}{Workers}\times {Days}}\times 100$$

for *i* days, *j* census tracts. An example interpretation of a rate of “10 exposures per 100 workers in 2010” could be “there were, on average, 10 exposures to occupational heat per day for every 100 workers in 2010”. We also assessed the number of worker-days of exposure across various temporal levels. These estimates could be aggregated to the county, state, region, and nationwide level (which could be further broken down by major SOC group) to show disproportionate burdens. Yearly estimates by census tract, county, state, and nationwide are publicly available at https://doi.org/10.5281/zenodo.15199026.

#### Assessing sociodemographic disparities

To test our hypothesis that marginalized populations of workers are disproportionately burdened by hazardous heat exposure at work, we ran four separate Poisson generalized additive models (GAM) using the yearly census tract-level percentage of racial and ethnic minority groups, low-income individuals, individuals with low educational attainment, and foreign-born individuals as the main, independent covariate of each model (using a smoothing spline with 4 degrees of freedom) and the estimated number of yearly worker-days exposed to occupational heat from 2010 to 2019 as the main outcome for each model. We included an offset term for the total number of residents in a census tract. We adjusted our models for the yearly, census tract-level percentage of individuals <16 years old, individuals >64 years old, and individuals who are unemployed, in the armed forces, or not in the labor force to control for individuals unlikely to be working. Models included a categorical variable for year to account for temporal autocorrelation and the census tract centroid’s latitude/longitude (tensor product with 40 degrees of freedom) to account for spatial autocorrelation.

We ran an additional regression model using the aforementioned four racial and ethnic subgroups (i.e., Hispanic, non-Hispanic Black, non-Hispanic Asian, and all other racial and ethnic minority subgroups) as the main independent covariates to assess whether specific minority groups may be highly exposed to occupational heat.

## Results

### Summary

**Fig. 3:**
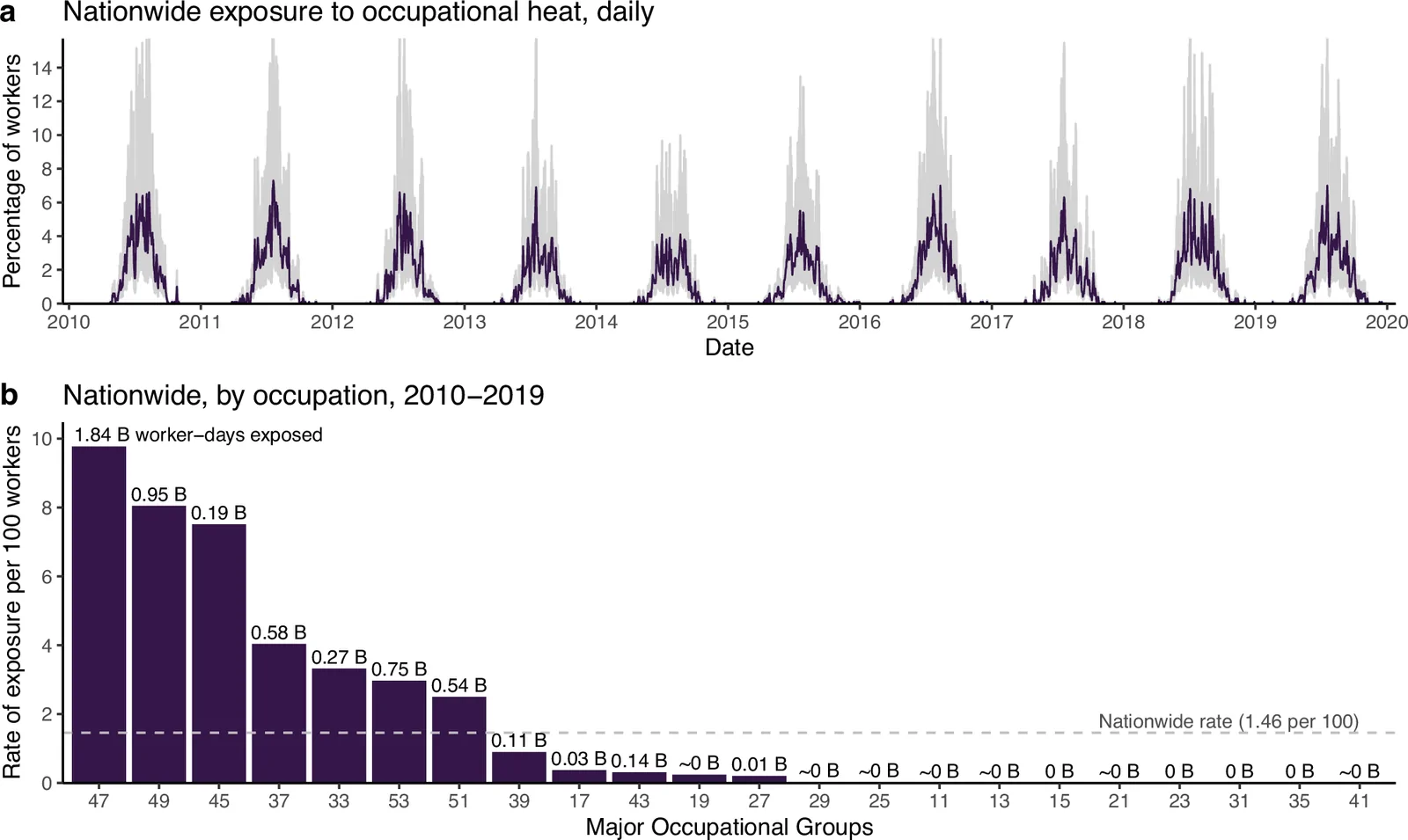
Trends in occupational heat exposure from 2010 to 2019. **a** Nationwide, daily percent of workers exposed to occupational heat. Gray, shaded areas represent uncertainty in estimates, where the lower limit reflects the unlikely, best case scenario (where all census tracts are set at their 2.5th percentile of daily occupational heat exposure) and the upper limit reflects the unlikely, worst case scenario (where all census tracts are set at their 97.5th percentile of daily occupational heat exposure). **b** Nationwide rate by major Standard Occupational Classification (SOC) codes across 2010–2019. Text labels above bars reflect total number of worker-days exposed to occupational heat, in billions. Major Occupational Groups: 11—Management Occupations; 13—Business and Financial Operations Occupations; 15—Computer and Mathematical Occupations; 17—Architecture and Engineering Occupations; 19—Life, Physical, and Social Science Occupations; 21—Community and Social Service Occupations; 23—Legal Occupations; 25—Educational Instruction and Library Occupations; 27—Arts, Design, Entertainment, Sports, and Media Occupations; 29—Healthcare Practitioners and Technical Occupations; 31—Healthcare Support Occupations; 33—Protective Service Occupations; 35—Food Preparation and Serving Related Occupations; 37—Building and Grounds Cleaning and Maintenance Occupations; 39—Personal Care and Service Occupations; 41—Sales and Related Occupations; 43—Office and Administrative Support Occupations; 45—Farming, Fishing, and Forestry Occupations; 47—Construction and Extraction Occupations; 49—Installation, Maintenance, and Repair Occupations; 51—Production Occupations; 53—Transportation and Material Moving Occupations.

Between 2010–2019, the estimated daily percent of workers exposed to hazardous heat conditions ranged from 0 to 7.3% of workers (Fig. 3a). Nine census tracts in Rio Arriba County, New Mexico in 2016 were considered to have 0% of workers exposed due to a lack of employment information (0.001% of tract-years). There were an estimated 5.44 billion worker-days of heat exposure in the US (Supplementary Table B1). This equated to 1.46 exposure events per 100 workers each day. July accounted for 33.5% (1.82 billion worker-days) of exposures, with monthly rates ranging between 4.84 to 8.93 exposure events per 100 workers each day in July (Supplementary Table B2).

Construction and Extraction workers (major SOC code 47-0000) were estimated to be the most highly exposed occupation, accounting for 33.9% (1.84 billion worker-days) of exposures despite making up 5.1% of workers in the United States (Fig. 3b). Construction and Extraction (9.77 per 100 workers; 47-0000), Installation, Maintenance, and Repair (8.05 per 100 workers; 49-0000), and Farming, Fishing, and Forestry occupations (7.51 per 100 workers; 45-0000) had the highest exposure rates from 2010–2019. Building and Grounds Cleaning and Maintenance (4.04 per 100 workers; 37-0000), Protective Service (3.32 per 100 workers; 33-0000), Transportation and Material Moving (2.97 per 100 workers; 53-0000), and Production occupations (2.50 per 100 workers; 51-0000) also had substantially higher exposure rates than the national average (1.46 per 100 workers).

Heat exposure varied by region (Supplementary Table B3). Workers in the US South make up estimated 36.5% of all workers but accounted for 81.7% (4.44 billion worker-days) of exposures. The average rate of exposure in the US South was 3.26 exposures per 100 workers each day from 2010 to 2019. Heat exposure in the Midwest, West, and Northeast were similar and below the nationwide average.

### Spatiotemporal variation in occupational heat exposure

**Fig. 4:**
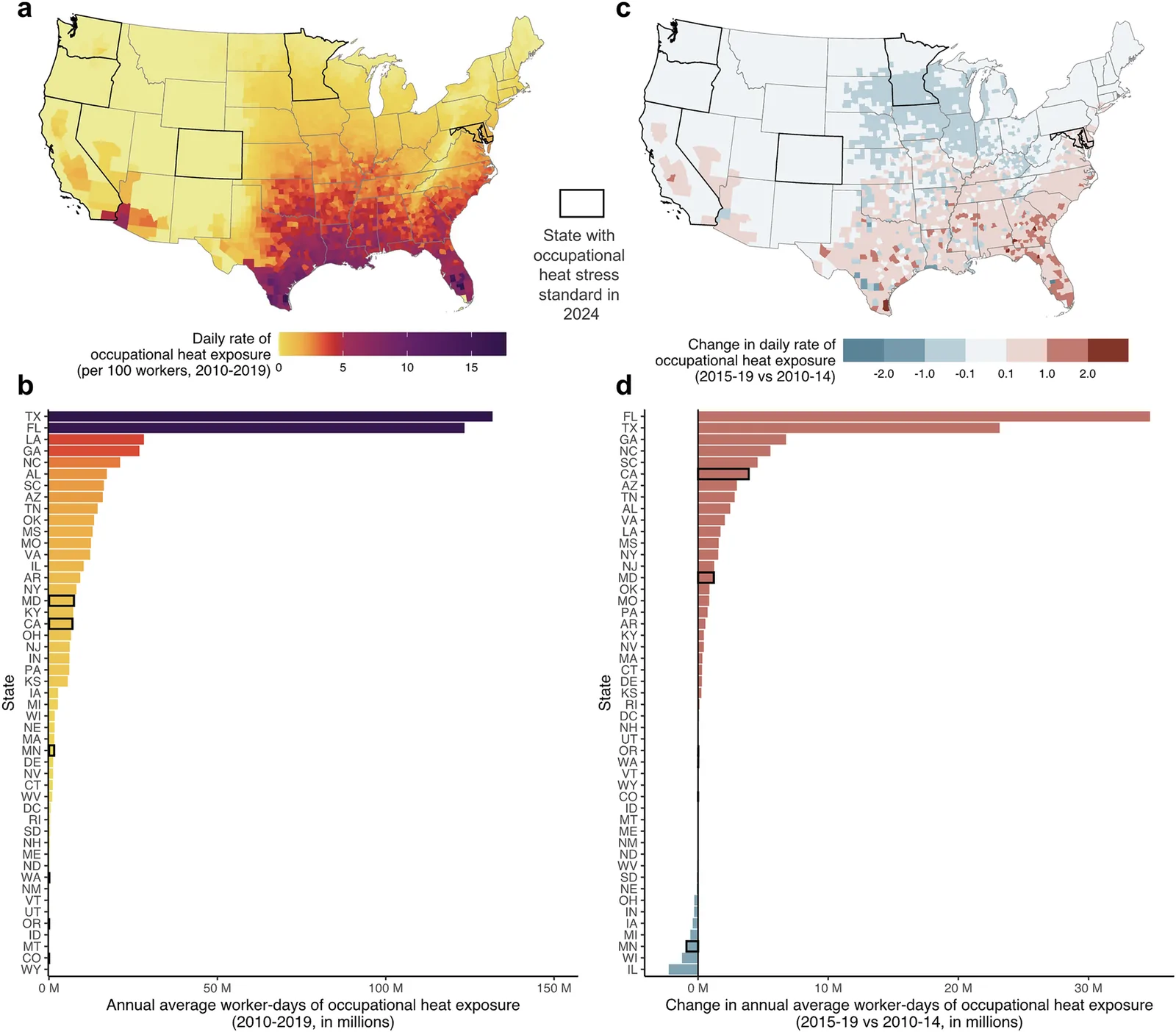
County-level occupational heat exposure and rate changes from 2010 to 2019. **a** Average rate of occupational heat exposure per 100 workers by county across all days between 2010 and 2019. **b** The average number of worker-days exposed to occupational heat each year between 2010 and 2019 broken down by each state. **c** The county-level difference between the annual average rate of occupational heat exposure per 100 workers in 2015–2019 and 2010–2014. **d** The difference between the annual average number of worker-days exposed to occupational heat in 2019–2014 and 2010–2014, broken down by each state.

There is spatial variation in hazardous heat exposure (Fig. 4a). The median county in the US had an average of 0.96 exposures per 100 workers each day from 2010 to 2019 (IQR: 0.15–2.88). Five percent of counties had an average daily rate of at least 5.83 exposures per 100 workers, and the maximum daily rate was 17.7 exposure per 100 workers in Hendry County, Florida. Workers in Louisiana and Florida had the highest exposure rates (Supplementary Table B4 and Fig. B9). Workers in Texas and Florida accounted for 46.9% of exposures annually (255 million worker-days per year) (Fig. 4b). Workers in Florida also saw the greatest increase in the daily rate of occupational heat exposure across the 2010s (Fig. 4c). There was an annual increase of 34.7 million worker-days of occupational heat exposure from 2010–2014 to 2015–2019 in Florida (Fig. 4d). In total, 31 other states also saw an increase in occupational heat exposure (in particular Georgia, North Carolina, and South Carolina). On the other hand, 16 states saw a decrease, particularly those in the Midwest (Illinois, Wisconsin, Minnesota, Michigan, and Iowa), likely driven by a decline in the percentage of days with high levels of heat (Supplementary Fig. B4) rather than decline in the percentage of outdoor workers (Supplementary Fig. B5).

### Sociodemographic disparities in occupational heat exposure

**Fig. 5:**
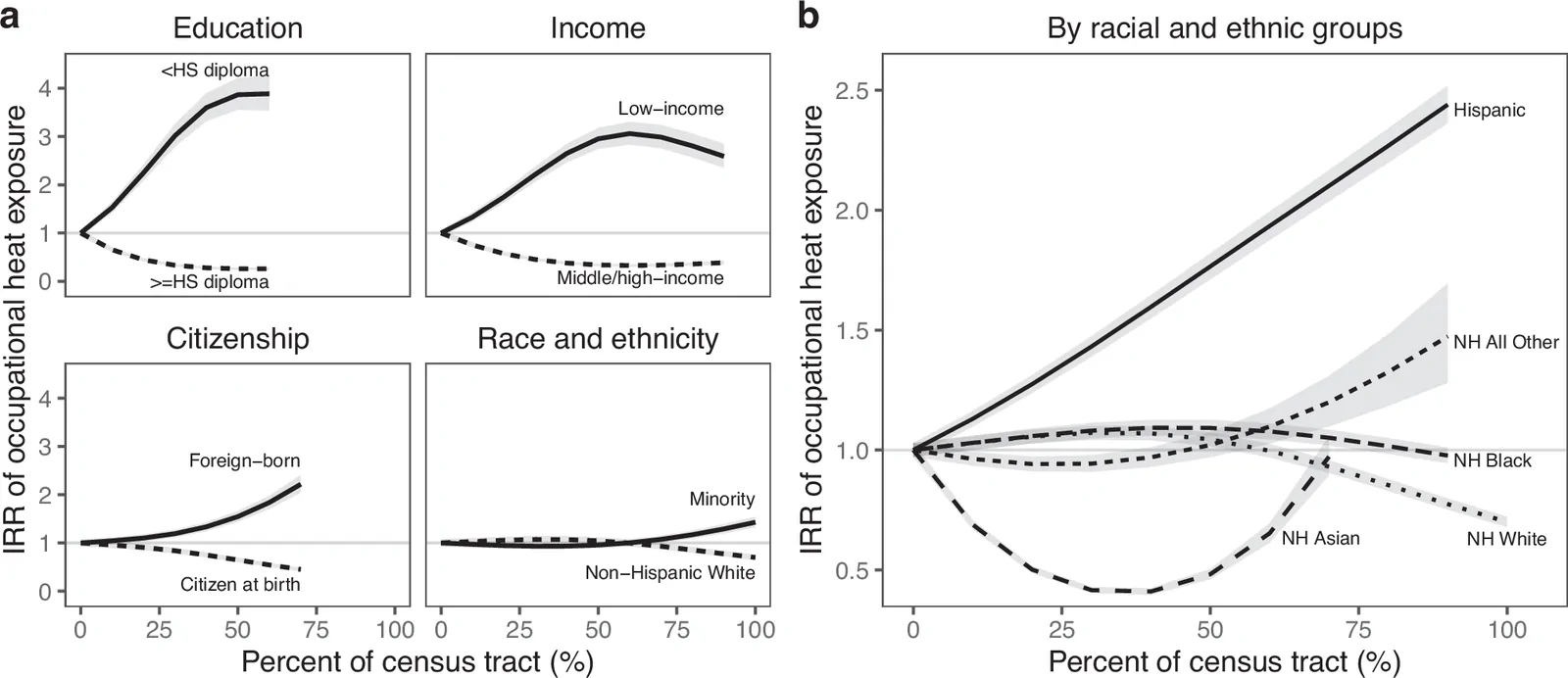
Census tract-level, sociodemographic disparities to occupational heat exposure across 2010–2019. Five, separate, Poisson generalized additive models (GAMs) were run with the census-tract level, yearly number of worker-days exposed to occupational heat as the outcome for each model. The main independent variables of interest were the census tract-level percentage of (**a**) low-income individuals, individuals without a high school diploma, racial and ethnic minority groups, and the foreign-born population, as well as broken down by race and ethnicity for (**b**) Hispanic, non-Hispanic Black, non-Hispanic Asian, non-Hispanic White, and all other non-Hispanic racial and ethnic minorities who do not self-identify as non-Hispanic White. Each GAM included the yearly census tract-level population as an offset term and the main sociodemographic characteristic using a smoothing spline with 4 degrees of freedom. Models also included the census tract-level percentage of individuals who are <16 years old, >64 years old, and unemployed/in armed forces/not in labor force, as well as a categorical variable for year to account for temporal autocorrelation and the census tract centroid’s latitude/longitude (tensor product with 40 degrees of freedom) to account for spatial autocorrelation. Note: IRR incidence rate ratio, NH non-Hispanic, HS high school.

Leveraging yearly, census tract-level estimates of occupational heat exposure rate, we assessed whether marginalized populations of workers are disproportionately burdened by occupational heat exposure during 2010–2019 using separate Poisson generalized additive models. After controlling for year, tract centroid, and the census tract-level percentage of individuals who <16 years old, >64 years old, or unemployed/in the armed forced/not in the labor force, we found that census tracts with a majority of individuals without a high school diploma were associated with a 3.5-fold increase in the rate of occupational heat exposure compared to tracts where all individuals have a high school diploma (Fig. 5a). Disparities were also observed among low-income individuals, racial and ethnic minority groups, and foreign-born individuals (around a 1.3- to 2.5-fold increase). Breaking down the associations by racial and ethnic minority subgroups, we observed that census tracts with a majority of Hispanic individuals were associated with a 2.5-fold increase in the rate of occupational heat exposure compared to census tracts without any Hispanic individuals (Fig. 5b). Some disparities were also observed among non-Hispanic Black individuals and all other racial and ethnic minority individuals who are not Hispanic, Black, or Asian. We observed an inverse association for non-Hispanic Asian.

### Sensitivity analysis

We estimated occupational heat exposure assuming all workers are acclimated to heat. Under this assumption, daily occupational heat exposure incidence varied from 0% to 2.5% of workers (Supplementary Fig. B6). There were 0.42 billion worker-days of heat exposure, equivalent to an acclimated rate of 0.11 exposures per 100 workers each day (Supplementary Fig. B8a). While Construction and Extraction occupations still had the largest number of worker-days exposed, these occupations had the second highest exposure rate assuming acclimatization (0.68 exposures per 100 workers). Installation, Maintenance, and Repair occupations had the highest rate (0.85 exposures per 100 workers) (Supplementary Fig. B8c).

## Discussion

Outdoor workers likely experienced an estimated 1.46 heat exposures events per 100 workers every day from 2010 to 2019. There was substantial spatiotemporal and occupational variation in estimated heat exposure. Rates peaked during July (ranging from 4.8 to 8.9 daily exposures per 100 workers). Workers in the US South (particularly in Texas, Louisiana, and Florida), and in construction/extraction occupations were the most highly exposed to heat. Marginalized populations of workers were disproportionately exposed, particularly those who are low-income, have low educational attainment, are Hispanic, or foreign-born.

There is a lack of previous work that estimates the incidence of occupational heat exposure. No previous studies characterized hazardous high heat exposure at the census tract level nor at a temporal scale as fine as daily, monthly, or yearly. One nationwide US study did estimate worker exposure to heat between 1971 and 2000 at the county level and provided exposure projections in 2036–2065 assuming greenhouse emissions don’t change [18]. This previous study estimated 315 million worker-days of exposure per year between 1971 and 2000 and projected to 1.4 billion in 2036–2065. Our approach estimated around 544 million worker-days of exposure per year between 2010 and 2019, placing our estimation firmly between their historical and projected estimates.

Most surveillance efforts have focused on characterizing heat-related injuries and illness. Nationally, there were 344 heat-related deaths from 2011 to 2019 [19], roughly equivalent to 0.02 deaths per 100,000 workers. For reference, there were 3.5 deaths per 100,000 workers for all types of fatalities in 2019 [20]. For injuries and illnesses, there were 31,560 cases from 2011 to 2019, equivalent to rate of 2.3 cases per 100,000 workers [21]. Meanwhile, we estimated around 1460 hazardous heat exposures per 100,000 workers. This discrepancy highlights how surveillance of worker illnesses and injuries is not optimal and may substantially under-characterize the true burden of occupational heat exposure on population health, particularly on other chronic endpoints of heat exposure such as kidney disease [1].

While occupational heat exposure did not vary substantially year-to-year nationally, temporal trends varied substantially by US region. Counties in the US Southwest and Southeast, particularly Florida and Texas, saw the largest increase in the population of workers exposed to heat, which appeared due to a combination of an increasing number of days with high heat and increasing proportion of outdoor workers. Of the top ten states with increasing occupational heat exposure, only one has a state-level occupational heat stress standard as of 2024 (California). Meanwhile, none of top three states with increasing exposure (Florida, Texas, and Georgia) have an OSHA-approved state plan. Without a state plan to implement an occupational heat stress standard [22] that would provide basic measures to deal with high heat, such as access to rest, cool water, and shade [23], employers in these three states would not be legally obligated to take these proactive measures to protect their workers unless federal OSHA passes its own heat standard. Further, both Florida and Texas both have adopted state-level policies expressly banning local governments from establishing protections for outdoor workers in extreme heat [24, 25].

Consistent with previous literature that shows marginalized workers are disproportionately burdened by occupational hazards [26–29], we found similar disparities to occupational heat exposure. Few studies have demonstrated occupational heat disparities. This is not because there is evidence to the contrary, but rather because this topic is understudied. We found that occupational heat exposure may most disproportionately impact low-income individuals. A non-peer reviewed analysis of workers compensation injury reports in California from 2001-2018 showed that the lowest-paid workers experienced around five times heat-related injuries as the highest-paid workers [30]. We observed that racial and ethnic minority groups are disproportionately burdened, particularly Hispanic and Black individuals, which is consistent with both nationwide and state-specific studies across varying time periods ranging from 1999 to 2019 [7, 21, 31]. We also observed that individuals with low educational attainment are disproportionately burdened, although there are a lack of studies supporting or contrasting this finding. That we found disparities among foreign-born workers is not surprising given their overrepresentation in occupations with high occupational heat exposure (e.g., construction, agriculture) [31].

Beyond potentially being the most exposed, the health effects of occupational heat on marginalized workers may be further compounded by factors during and outside of work. During work, marginalized workers may lack the power and resources to protect themselves from the effects of heat (e.g., lack of suitable workplace controls or poor worker’s compensation) [32]. Outside of work, marginalized workers may be doubly exposed to environmental heat, with racial and ethnic minorities and low-income individuals disproportionately burdened by urban heat island effects and a lack of access to air conditioning [33–35], potentially limiting their ability to recover from occupational heat exposure.

### Limitations, strengths, and future research

The degree to which our estimates of occupational heat exposure are biased or valid cannot be determined. However, we made specific analytic decisions that balance under- and over-estimation to avoid strong influence in either direction. A large limitation to our approach is characterizing occupational heat exposure among outdoor workers only. While outdoor workers represent a large portion of those exposed, overlooking indoor workers may underestimate those exposed and potentially misclassify the most exposed areas of the United States. We may have further underestimated exposure by using average WBGT levels, rather than maximum levels, to determine overexposure since temperatures typically peak in the middle of the day when outdoor workers are more likely to be working. We used average levels since there is a lack of information of typical work times for detailed SOC groups. We also did not consider clothing adjustment values, which likely underestimates exposure for the portion of workers who work in heavier or less-breathable garments.

On the other hand, we overestimate exposure by determining exposure according to unacclimatized workers since some unknown portion of workers are likely acclimatized to heat. We also likely overestimated when people are working on weekends. This was determined if a detailed SOC code typically involves >40 h of work per week or has an irregular work schedule; however, some unknown portion of workers likely work >40 h per week during weekdays and others who have an irregular work schedule may only work during the colder or hotter months. Furthermore, while we assume that the vast majority of outdoor workers in most industries cannot avoid extreme heat days (e.g., agriculture, construction, protective services), some work sites likely alter working hours on particularly hot days, which could slightly bias our estimates high.

Overall, caution should be taken with interpretation of our exposure characterization. Our approach estimated workers who are potentially exposed to excessive levels of workplace heat, and thus, may be at risk of heat stress. This is not an estimation of personal heat stress risk. Our approach lacked other key, detailed information on workplace characteristics that could influence personal risk, such as access to water and cooling throughout the day. Additionally, developing these exposure estimates required the integration of multiple, disparate data sources, each with their own sources of error, that may limit the precision of the estimates. We conducted a Monte Carlo simulation approach to quantify some of this variability, and future work could link these estimates to trends in adverse health outcomes (e.g., mortality data) to test the validity of our approach. Our geospatial approach is also limited by the modifiable areal unit problem; we used arbitrary administrative boundaries (i.e., census tract, county, state) to assess spatial patterns in heat exposure, and using different spatial boundaries might result in different exposure patterns.

Alongside these limitations are some strengths to our approach. We incorporated important data on heat-related workplace characteristics to improve the robustness of our findings (i.e., outdoor work, long/irregular work schedules, metabolic rate, and work/rest allocation). The construction of these workplace characteristic metrics were reviewed by experts in occupational health to ensure they appeared valid; however, specific adjustments were not made to ensure reproducibility of our analysis. Future research may consider making targeted adjustments to these metrics to further increase validity in this approach. Overall, our findings also appear reliable. For example, we found that construction/agricultural occupations and marginalized workers were the most exposed to occupational heat, which is consistent with previous research on heat-related injuries and illnesses.

Future work seeking to improve our exposure assessment approach could incorporate measures of indoor WBGT levels to more wholistically assess the magnitude of workers exposed to heat as well as information on workplace cooling. Our current estimates can also improve epidemiological and health impact studies of heat-related disease and mortality to better characterize the burden of heat. Current approaches do not consider how workplace exposures may alter local susceptibility to the effects of heat. This exposure misclassification may be underestimating heat-related health burdens in areas with high workplace heat exposure, and may be conversely overestimate burdens in areas with low workplace exposure [36, 37], particularly for studies of future climate projections [38, 39].

### Policy implications

Characterizing spatial and temporal trends of occupational heat exposure can support targeted enforcement and consultation campaigns by both federal and state OSHA plans. While recent approaches by OSHA (through the 2022 National Emphasis Program) target specific industries that are expected to have the highest heat-related exposures to encourage early interventions that prevent illnesses and deaths [40], integrating spatiotemporal information of potential exposures can better target industries in particular communities in the US. This spatiotemporal information can also inform non-occupational prevention efforts of state- and local-health departments to target surveillance efforts and healthcare resources towards communities with likely high occupational exposures and low socioeconomic resources.

## Conclusion

The incidence of occupational heat exposure, its disproportionate burden on marginalized workers, and the increasing impact of climate change suggests a critical and urgent need for occupational regulation and robust surveillance of heat exposure to reduce heat-related injuries, illnesses, and fatalities.

## Supplementary information

Supplementary Information

## Data Availability

The final, simulated occupational heat incidences by county can be explored and visualized at https://sph-umich.shinyapps.io/work-heat-2010-2019/. Yearly breakdowns by census tract, county, state, and nationwide are available at https://doi.org/10.5281/zenodo.15199026. For daily exposure rate estimates, contact the corresponding author (Abas Shkembi; email: ashkembi@umich.edu). All data underlying the analysis are publicly available. Census tract-level major employment counts and sociodemographic compositions are available from the ‘tidycensus‘ R package (https://cran.r-project.org/web/packages/tidycensus/index.html). County-level wet bulb globe temperature levels were previously published by Spangler et al., 2020 (https://doi.org/10.6084/m9.figshare.19419836). Metropolitan/nonmetropolitan-level detailed employment counts are available by the Bureau of Labor Statistics Occupational Employment and Wage Statistics (https://www.bls.gov/oes/tables.htm). Workplace characteristics are made publicly available from version 24.1 of the Occupational Information Network (https://www.onetcenter.org/db_releases.html).

## Code availability

Replication code required to reproduce the results are maintained in the following GitHub repository: https://github.com/abasshkembi/work-heat.
