# Supplementary material for: Occupational heat exposure incidence and sociodemographic disparities for outdoor workers in the United States, 2010–2019
Source: J Expo Sci Environ Epidemiol. 2025 Oct 9;36(6):1045–53. doi: 10.1038/s41370-025-00813-y (PMC13727679; doi:10.1038/s41370-025-00813-y)
Supplement: Supplementary file 1 [file 41370_2025_813_MOESM1_ESM.docx]

*Supplemental Materials for*

**Occupational heat exposure incidence and sociodemographic disparities for outdoor workers in the United States, 2010-2019**

Abas Shkembi^1,*^, Devon Payne-Sturges^1^, Sung Kyun Park^1,2^, Richard L. Neitzel^1^

**Affiliations:**

^1^Department of Environmental Health Sciences, University of Michigan School of Public Health, Ann Arbor, MI, USA

^2^Department of Epidemiology, University of Michigan, Ann Arbor, MI, USA

**^*^Correspondence:**

Abas Shkembi, Department of Environmental Health Sciences, University of Michigan School of Public Health, 1415 Washington Heights, Ann Arbor, MI, USA 48109 (e: [ashkembi@umich.edu](mailto:ashkembi@umich.edu))

**___________________**

**Table of Contents**

**Appendix A.** Supplemental Methods

**Appendix B.** Supplemental Tables and Figures

**Appendix A. Supplemental Methods**

**Rationale for using ACGIH screening criteria for unacclimatized workers.** No nationwide regulations for occupational heat exposure exist in the US, although six states (California, Colorado, Minnesota, Oregon, Washington, and Marlyand) have passed state-level standards as of 2024. Several organizations have developed guidelines to protect workers from the effects of occupational heat, including (i) the American Conference of Governmental Industrial Hygienists (ACGIH), a private, non-profit, non-governmental organization; (ii) the National Institute of Occupational Safety and Health (NIOSH), a governmental research agency a part of the United States Centers for Disease Control that sets recommendations for the regulatory agency, the Occupational Safety and Health Administration; (iii) the International Organization for Standard (ISO), a non-governmental organization that sets standards comprised of a majority of nations worldwide; and (iv) the American Industrial Hygiene Association (AIHA), a professional, non-profit association. We used guidelines set by ACGIH to determine exposure to occupational heat.

ACGIH is the world-leading organization in setting recommendations of guidelines for controlling workplace hazards. The guidelines represent conditions where nearly all workers may be continuously exposed to a given workplace hazard without adverse health effects, as ACGIH does not consider economic feasibility when determining guidelines. Therefore, guidelines set by ACGIH represent the most up-to-date and scientifically rigorous recommendations for occupational heat that are most protective of worker health.

ACGIH provides guidelines for both acclimatized and unacclimatized workers. Acclimatized workers are those who have gradually developed a tolerance to high levels of heat over a few weeks. The majority of heat-related illnesses occur during a worker’s first week when workers are unacclimatized, are returning back to work after some time off, or when temperatures increase above a level where a worker is already acclimatized [1]. To reflect the adverse health effects of unacclimatization such that our estimates are more inclusive of all workers who could be at risk of heat-related injuries and illnesses, our main results are presented according to unacclimatized workers. As a sensitivity, we compared our findings assuming all workers are acclimatized (see **Appendix B**).

**Determining exposure to occupational heat.** ACGIH recommends an on-site, detailed assessment of wet-bulb globe temperatures (WBGT) and worker characteristics to determine whether a worker may be overexposed to heat. However, when such detailed assessment cannot be completed, ACGIH also provides screening criteria. We utilized these screening criteria set in 2023 to determine which workers are overexposed to occupational heat [2].

| **Table A1.** Screening criteria using WBGT (in Celsius) for unacclimatized workers set by ACGIH in 2023. From: *2023 TLV®s and BEI®s, © ACGIH 2023.* | | | | |
| --- | --- | --- | --- | --- |
|  | **Metabolic rate** | | | |
| **Work/rest allocation** | **Light** | **Moderate** | **Heavy** | **Very Heavy** |
| **75 to 100%** | 28 | 25 | *24^a^* | *24^a^* |
| **50 to 75%** | 28.5 | 26 | 24 | *24^a^* |
| **25 to 50%** | 29.5 | 27 | 25.5 | 24.5 |
| **0 to 25%** | 30 | 29 | 28 | 27 |
| ^a^ACGIH does not originally provide screening WBGT values for heavy and very heavy work among those with a high work allocation because of the unsustainability of the work. We set these values to the lowest WBGT value in the table (24°C) | | | | |

**Supplemental Table A2.** List of each variable used to construct occupational heat exposure rates across three steps (Step 1: Data integration; Step 2: Monte Carlo simulation; and Step 3: Poststratification).

| **Step** | **Variable** | **Data source** | **Temporal scale** | **Spatial scale** | **Data description** | **Variable definition** |
| --- | --- | --- | --- | --- | --- | --- |
| 1 | Wet-bulb globe temperature | Spangler et al. (2020) [3] | Daily, 2000-2020 | County | Ambient wet-bulb globe temperature (estimated in °C) for every county in the contiguous United States each day from January 1, 2000 to December 31, 2020 | Exposure was classified according to Table A1 |
| 1, 2 | Outdoor work | O*NET v24.1 [4] | Decadal, 2010-2019 | Nationwide | An estimate of the “Frequency Required to Work Outdoors, Exposed to Weather” work context variable (element ID #4.C.2.a.1.c), reflecting “How often does this job require working outdoors, exposed to all weather conditions?” (scale of 0-100) for every detailed SOC group | Converted to probability of working outdoors on a given day (0-100%) |
| 1 | Irregular work hours | O*NET v24.1 [4] | Decadal, 2010-2019 | Nationwide | An estimate of the “Duration of Typical Work Week” work context variable (element ID #4.C.3.d.8), reflecting “Number of hours typically worked in one week” (scale of 0-100) for every detailed SOC group | If either the first value was >50 or the second value was >25, detailed SOC group was considered as working irregular hours |
|  |  | O*NET v24.1 [4] | Decadal, 2010-2019 | Nationwide | An estimate of the “Work Schedules” work context variable (element ID #4.C.3.d.4), reflecting “How regular are the work schedules for this job?” (scale of 0-100) for every detailed SOC group |  |
| 1 | Metabolic rate | O*NET v24.1 [4] | Decadal, 2010-2019 | Nationwide | “Light” - an estimate of the “Time Spent in Body Positions - Spend Time Sitting” work context variable (element ID #4.C.2.d.1.a), reflecting “How much does this job require sitting?” (scale of 0-100) for every detailed SOC group | Assigned into one of four categories, depending on which value was largest:  [“Light”, “Moderate”, “Heavy”, “Very Heavy”] |
|  |  | O*NET v24.1 [4] | Decadal, 2010-2019 | Nationwide | “Moderate” - an estimate of the “Time Spent in Body Positions, Spend Time Standing” work context variable (element ID #4.C.2.d.1.b), reflecting “How much does this job require standing?” (scale of 0-100) for every detailed SOC group |  |
|  |  | O*NET v24.1 [4] | Decadal, 2010-2019 | Nationwide | “Heavy” - an average estimate, for every detailed SOC group, of the   - “Time Spent in Body Positions, Spend Time Standing” work context variable (element ID #4.C.2.d.1.b), reflecting “How much does this job require standing?” (scale of 0-100), and the - “Time Spent in Body Positions, Spend Time Using Your Hands to Handle, Control, or Feel Objects, Tools, or Controls” work context variable (element ID #4.C.2.d.1.g), reflecting “How much does this job require using your hands to handle, control, or feel objects, tools or controls?” (scale of 0-100) |  |
|  |  | O*NET v24.1 [4] | Decadal, 2010-2019 | Nationwide | “Very Heavy” - an estimate of the “Time Spent in Body Positions, Spend Time Walking and Running” work context variable (element ID #4.C.2.d.1.d), reflecting “How much does this job require walking and running?” (scale of 0-100) for every detailed SOC group |  |
| 1 | Work/rest allocation | O*NET v24.1 [4] | Decadal, 2010-2019 | Nationwide | An estimate of the “Time Spent in Body Positions - Spend Time Sitting” work context variable (element ID #4.C.2.d.1.a), reflecting “How much does this job require sitting?” (scale of 0-100) for every detailed SOC group | Calculated \|50 - *value*\|, classified new value as a percentage, and assigned final value into four categories:  [0-25%, 25-50%, 50-75%, 75-100%] |
| 1 | Detailed employment counts | 2019 BLS OEWS [5] | Decadal, 2010-2019 | Metropolitan/  Nonmetropolitan area | Metropolitan/nonmetropolitan area employment counts of 840 detailed SOC groups from 2010-2019 | Number of civilian, non-military, employed individuals |
| 3 | Major employment counts | US American Community Survey | Yearly, 2010-2019 | Census tract | Census tract-level employment counts of 22 major SOC groups from 2010-2019, aggregated from sex-specific estimates. For males, we extracted variables 5-37 from Table C24010. For females, we extracted variables 41-73 from Table C24010 | Number of civilian, non-military, employed individuals |

**Detailed workplace characteristic data.** An exhaustive list of all data sources, variables, definitions, and calculations is presented in **Table A2**. To determine whether a given occupation may be exposed to occupational heat, we leveraged data by detailed, 2010 Standard Occupational Classification (SOC) codes in the 2019 Occupational Information Network (O*NET, version 24.1) [4]. The O*NET database is made publicly available and developed by the US Department of Labor. The O*NET database provides estimates of various work characteristics using self-reported data collected by the Department of Labor. The data are provided by detailed, 2010, O*NET-SOC codes. The 9-digit, detailed O*NET-SOC codes were converted to 7-digit, detailed BLS SOC codes by aggregated the first 7 digits of the O*NET-SOC codes and taking the average value by detailed SOC code.

Specifically, we determined (i) the probability a detailed SOC group may be working outdoors, (ii) whether a detailed SOC group typically works long or irregular work schedules, and thus, might work on the weekend, (iii) the metabolic rate of the detailed SOC group, and (iv) allocation of work in a cycle of work and recovery by detailed SOC group.

***Figure A1.*** *Histogram by detailed SOC group (n=760) of the proportion of work-year outdoors*

*Probability of outdoor work.* We used the continuous “Frequency Required to Work Outdoors, Exposed to Weather” work context variable (element ID #4.C.2.a.1.c). Specifically, workers responded to the following question: “How often does this job require working outdoors, exposed to all weather conditions?”. Possible responses ranged from “Never” to “Everyday”. This estimate is provided on a scale of 0-100, which was rescaled to 0-250 to reflect the standard, 250-day work-year, as previously done by our study team [6]. We then converted the estimate back onto a scale of 0-1 to reflect the proportion of the standard work-year that a worker is working outdoors.

***Figure A2.*** *Bar chart by detailed SOC group (n=760) of weekend work*

*Long/irregular work schedules.* We used two variables to determine whether a detailed SOC group works long or irregular schedules, and thus, could reasonably work on the weekend. First, we used the continuous “Duration of Typical Work Week” work context variable (element ID #4.C.3.d.8). Specifically, workers responded to the following question: “Number of hours typically worked in one week”. Values ranged from 0-100. Possible responses were from “Less than 40 hours” (value of 0), “40 hours” (value of 50), and “More than 40 hours” (value of 100). We assigned a binary value of whether a detailed SOC group works more than 40 hours if the value was >50. Next, we used the continuous “Work Schedules” work context variable (element ID #4.C.3.d.4). Specifically, workers responded to the following question: “How regular are the work schedules for this job?”. Values ranged from 0-100. Possible responses were from “Regular (established routine, set schedule)” (value of 0), “Irregular (changes with weather conditions, production demands, or contract duration)” (value of 50), and “Seasonal (only during certain times of the year)” (value of 100). We assigned a binary value of whether a detailed SOC group works irregularly if the value was >25. If either the first value was >50 or the second value was >25, we considered the detailed SOC group as likely “weekend workers”.

*Metabolic rate.* We used four variables to determine metabolic rate. We assigned each detailed SOC group as “light”, “moderate”, “heavy”, or “very heavy” metabolic rate if one of the (i) time spent sitting, (ii) time spent standing, (iii) average time spent standing and using hands, and (iv) time spent walking/running has the highest possible value, respectively.

First, we used the continuous “Time Spent in Body Positions, Spend Time Sitting” work context variable (#4.C.2.d.1.a) for (i) light metabolic rate. Specifically, workers answered “How much does this job require sitting?”. Second, we used the continuous “Time Spent in Body Positions, Spend Time Standing” work context variable (#4.C.2.d.1.b) for (ii) moderate metabolic rate. Specifically, workers answered “How much does this job require standing?”. Third, we took the average of this previous variable and the continuous “Time Spent in Body Positions, Spend Time Using Your Hands to Handle, Control, or Feel Objects, Tools, or Controls” work context variable (#4.C.2.d.1.g) for (iii) heavy metabolic rate. Specifically, workers answered “How much does this job require using your hands to handle, control, or feel objects, tools or controls?”. Fourth, we used the continuous “Time Spent in Body Positions, Spend Time Walking and Running” work context variable (#4.C.2.d.1.d) for (ii) very heavy metabolic rate. Specifically, workers answered “How much does this job require walking and running?”. Each of the four factors had values ranging from 0-100 (from “Never” to “Continually or almost continually”). The factor that had the highest value among a detailed SOC group was assigned as the metabolic rate for that group.

*Work/rest allocation.* To determine the work vs recovery time, we again used the continuous “Time Spent in Body Positions, Spend Time Sitting” work context variable (#4.C.2.d.1.a). We considered time spent sitting as “recovery time”, and time spent not sitting as “work time”. Below is a table that breaks down the spread of 760 detailed SOC groups by metabolic rate and work/rest allocation.

| **Table A3.** Counts of detailed SOC groups (n=760) by metabolic rate and work/rest allocation | | | | |
| --- | --- | --- | --- | --- |
|  | **Metabolic rate** | | | |
| **Work/rest allocation** | **Light** | **Moderate** | **Heavy** | **Very Heavy** |
| **75 to 100%** | 0 | 101 | 76 | 3 |
| **50 to 75%** | 2 | 79 | 100 | 6 |
| **25 to 50%** | 160 | 8 | 45 | 1 |
| **0 to 25%** | 179 | 0 | 0 | 0 |

**Detailed employment count data by county.** We extracted 2019 metropolitan/nonmetropolitan area employment counts of detailed Standard Occupational Classification (SOC) codes from the US Bureau of Labor Statistics (BLS) Occupational Employment and Wage Statistics (OEWS) [5]. Metropolitan areas are counties grouped together based on common economic activities and commuting patterns around a city. Thus, some metropolitan areas are a single county, while others are a combination of a few counties. Nonmetropolitan areas are larger groups of many counties that do not have economic activity around a major city. We assumed that the spread of detailed SOC codes was the same for the counties within a metro/nonmetro area. These detailed SOC employment counts are not developed to be assessed in a time-varying manner, as the OEWS aggregates data across many years to develop their estimates. Thus, we used 2019 data to span across the years of analysis in this study (2010-2019). In 2018, SOC codes were updated from the 2010 SOC code version. We integrated all data to 2010 SOC codes since the majority of data used in this study used the 2010 SOC structure. Thus, we crosswalked 2018 detailed SOC codes from the OEWS to 2010 detailed SOC codes.

***Figure A3****. Boxplot of proportion exposed to occupational heat by county for 08-01-2010 by 50, 100, 200, 500, and 1000 MC iterations. (a) Median estimate, (b) 2.5^th^ percentile estimate, and (c) 97.5^th^ percentile estimate.*

**Monte Carlo simulation.** We estimated the proportion of workers exposed to occupational heat for every day from 2010-2019, broken down by county and major SOC group. This estimation was performed using Monte Carlo simulation. To determine the optimal number of iterations for the Monte Carlo (MC) simulation (balancing time for computation and bias of estimates), we tested different iteration counts for a random day in the study period (August 1, 2010). Specifically, we compared the median, 2.5^th^ percentile, and 97.5^th^ percentile of each major SOC-county estimate.

As shown in **Figure A3**, estimation of the median, 2.5^th^ percentile and 97.5^th^ percentile did not vary substantially past 200 MC iterations.

**Major employment count data by census tract for poststratification.** We extracted yearly breakdowns of the 22, non-military, 2010, major SOC employment counts by census tract from the 5-year, US American Community Survey (ACS) for poststratification of the major SOC-county estimates of exposure to the census tract-level. The estimates were extracted using the `tidycensus` package in R [7]. The estimates are further broken down by sex, so we aggregated the sex-specific estimates together. For males, we extracted variables 5-37 from Table C24010. For females, we extracted variables 41-73 from Table C24010.

**Assessment of analytical decisions on estimation bias.** Several analytical decisions and assumptions were required to integrate multiple data sources, each with their own sources of error. Below, we list the major analytical decisions and assumptions made and our assessment how each assumption would bias our estimates of the proportion of workers exposed to occupational heat.

1. **↓ Assess exposure among outdoor workers who are exposed to the sun only.**
   a. Environmental heat can place indoor workers in poorly, environmentally controlled conditions (e.g., no air conditioning).
   b. It can also impact outdoor workers who primarily work in covered/shaded conditions if there is a lack of wind.
   c. This decision likely underestimates exposure.
2. **↓ No clothing adjustment values.**
   a. Workers who have to perform job tasks in heavier or non-breathable garments will have increased risk of heat stress.
   b. ACGIH recommends adjusting WBGT levels based on the clothing a worker is in.
   c. This data is not available in the O*NET and was not considered.
   d. This decision likely underestimates exposure.
3. **↓ Using mean WBGT levels instead of maximum.**
   a. Heat typically peaks during the middle of the day when the sun is high.
   b. Maximum WBGT levels may be more indicative of risk of heat stress than mean levels.
   c. To our knowledge, there is a lack of publicly available information of working times by detailed SOC group, and the mean was used instead.
   d. This decision likely underestimates exposure.
4. **↑ Determine exposure based on screening criteria for unacclimatized workers.**
   a. Some unknown percentage of workers are likely already acclimatized to heat.
   b. This decision likely overestimates exposure.
5. **↑ Determine weekend work based on typical work week >40 hours or irregular work schedule.**
   a. Workers may work >40 hours a week but work all of those hours during weekdays.
   b. Workers may have an irregular work schedule but only work seasonally.
   c. This decision likely overestimates exposure.
6. **↔ Assume people work in the county that they live in.**
   a. In order to assign mean WBGT levels for each worker, we assume that the worker group works and lives in the same county.
   b. Some unknown percentage of workers work in a different county that they live in.
   c. Given county-level, mean WBGT levels are highly spatially autocorrelated between neighboring counties, this decision likely has a non-differential impact on estimates.
7. **↑↓ Determine work/rest allocation using time sitting vs standing.**
   a. For some jobs, time spent sitting is working (e.g., heavy equipment operator) while time spent standing is resting (e.g., waiter).
   b. By determining work/rest allocation using time sitting vs standing, our estimates may be biased high or low for some occupations.
8. **↑↓ Assume employment counts by detailed SOC groups did not vary day-to-day and year-to-year across the study analysis.**
   a. According to the Bureau of Labor Statistics, detailed SOC employment counts were not developed to be assessed in a time-varying manner, as the OEWS aggregates data across many years to develop their estimates.
   b. This likely overestimates exposures in some counties, while underestimating them in others.
9. **↑↓ Assume the spread of detailed SOC groups is the same between metropolitan/nonmetropolitan areas and counties.**
   a. We made this assumption in order to merge county-level, mean WBGT levels with detailed SOC group employment counts at the metropolitan/nonmetropolitan area-level.
   b. This likely overestimates exposures in some counties, while underestimating them in others.

**References**

1.	Tustin AW, Cannon DL, Arbury SB, Thomas RJ, Hodgson MJ. Risk Factors for Heat-Related Illness in U.S. Workers. J Occup Environ Med. 2018 Aug;60(8):e383–9.

2.	ACGIH. 2023 TLVs and BEIs Based on the Documentation of the Threshold Limit Values for Chemical Substances and Physical Agents. American Conference of Governmental Industrial Hygienists; 2023. 1–287 p.

3.	Spangler KR, Liang S, Wellenius GA. Wet-Bulb Globe Temperature, Universal Thermal Climate Index, and Other Heat Metrics for US Counties, 2000–2020. Sci Data. 2022 Jun 17;9(1):326.

4.	U.S. Department of Labor. O*NET 24.1 [Internet]. 2019 [cited 2025 Jan 13]. Available from: https://www.onetcenter.org/dictionary/24.1/excel/

5.	U.S. Bureau of Labor Statistics. Occupational Employment and Wage Statistics [Internet]. U.S. Department of Labor. 2020 [cited 2023 Dec 14]. Available from: https://www.bls.gov/oes/special-requests/oesm19ma.zip

6.	Shkembi A, Zelner J, Park SK, Neitzel R. Workplace Exposures Vary Across Neighborhoods in the US: Implications on Social Vulnerability and Racial/Ethnic Health Disparities. J Racial Ethn Health Disparities. 2024 Aug 30;

7.	Walker K, Herman M. tidycensus: Load US Census Boundary and Attribute Data as “tidyverse” and ’sf’-Ready Data Frames [Internet]. 2022. Available from: https://CRAN.R-project.org/package=tidycensus

**Appendix B. Supplemental Tables and Figures**

| **Table B1.** Estimated number of worker-days exposed to occupational heat and the associated rate of exposure per 100 workers by year across 2010-2019. | | | |
| --- | --- | --- | --- |
| **Year(s)** | **Number of worker-days exposed** | **Annual average worker count** | **Rate**  **per 100 workers** |
| 2010-2019 | 5,439,577,797 | 149,261,359 | 1.46 |
| 2010 | 593,851,370 | 141,996,548 | 1.67 |
| 2011 | 558,468,513 | 141,864,697 | 1.57 |
| 2012 | 484,064,112 | 143,435,233 | 1.35 |
| 2013 | 442,841,384 | 145,747,779 | 1.22 |
| 2014 | 402,085,757 | 148,001,326 | 1.09 |
| 2015 | 487,351,087 | 150,599,165 | 1.29 |
| 2016 | 626,773,848 | 152,726,700 | 1.64 |
| 2017 | 512,732,892 | 154,842,185 | 1.32 |
| 2018 | 687,246,523 | 155,888,980 | 1.76 |
| 2019 | 644,162,311 | 157,510,982 | 1.64 |

| **Table B2.** Estimated number of worker-days exposed to occupational heat and the associated rate of exposure per 100 workers by month of year across 2010-2019. | | |
| --- | --- | --- |
| **Month** | **Number of worker-days exposed** | **Rate**  **per 100 workers** |
| January | 0 | 0 |
| February | 210,168 | <0.01 |
| March | 1,204,618 | <0.01 |
| April | 24,787,930 | 0.08 |
| May | 237,591,241 | 0.76 |
| June | 1,043,897,379 | 3.36 |
| July | 1,823,661,135 | 5.86 |
| August | 1,419,834,948 | 4.57 |
| September | 729,351,458 | 2.35 |
| October | 141,352,710 | 0.46 |
| November | 14,858,611 | 0.05 |
| December | 2,827,599 | 0.01 |

| **Table B3.** Estimated number of worker-days exposed to occupational heat and the associated rate of exposure per 100 workers by US region across 2010-2019. | | | |
| --- | --- | --- | --- |
| **Region** | **Number of worker-days exposed** | **Annual average worker count** | **Rate**  **per 100 workers** |
| South | 4,443,268,507 | 54,539,873 | 3.26 |
| Midwest | 515,704,286 | 32,634,756 | 0.63 |
| Northeast | 237,896,368 | 27,265,556 | 0.35 |
| West | 242,708,635 | 24,821,174 | 0.28 |

| **Table B4.** Estimated number of worker-days exposed to occupational heat and the associated rate of exposure per 100 workers by US state across 2010-2019. | | | |
| --- | --- | --- | --- |
| **State** | **Number of worker-days exposed** | **Annual average worker count** | **Rate**  **per 100 workers** |
| Louisiana | 281,762,730 | 2,016,662 | 5.59 |
| Florida | 1,234,081,040 | 8,933,278 | 5.53 |
| Mississippi | 129,483,372 | 1,218,417 | 4.25 |
| Texas | 1,317,433,195 | 12,529,298 | 4.21 |
| Alabama | 171,514,392 | 2,061,804 | 3.33 |
| Oklahoma | 133,910,505 | 1,737,427 | 3.08 |
| South Carolina | 162,755,495 | 2,154,500 | 3.02 |
| Arkansas | 92,849,266 | 1,276,575 | 2.91 |
| Georgia | 268,576,466 | 4,576,637 | 2.35 |
| Arizona | 159,830,400 | 2,946,033 | 2.17 |
| Tennessee | 144,312,236 | 2,978,697 | 1.94 |
| North Carolina | 211,083,338 | 4,528,293 | 1.86 |
| Missouri | 124,660,623 | 2,852,597 | 1.75 |
| Kansas | 55,671,681 | 1,416,810 | 1.57 |
| Kentucky | 71,473,475 | 1,927,291 | 1.48 |
| Virginia | 122,425,723 | 4,047,492 | 1.21 |
| Delaware | 12,396,370 | 441,185 | 1.12 |
| Maryland | 74,077,533 | 3,014,551 | 0.98 |
| Indiana | 60,543,359 | 3,102,322 | 0.78 |
| Iowa | 26,957,077 | 1,588,896 | 0.68 |
| Nebraska | 16,598,704 | 979,071 | 0.68 |
| Illinois | 103,114,462 | 6,145,483 | 0.67 |
| District of Columbia | 5,293,826 | 349,550 | 0.61 |
| New Jersey | 61,745,483 | 4,347,326 | 0.57 |
| West Virginia | 9,839,545 | 748,218 | 0.53 |
| Ohio | 65,468,393 | 5,455,515 | 0.48 |
| Pennsylvania | 60,119,904 | 6,078,793 | 0.40 |
| Nevada | 11,902,334 | 1,325,037 | 0.36 |
| New York | 81,271,300 | 9,338,225 | 0.35 |
| South Dakota | 3,240,564 | 434,801 | 0.30 |
| Rhode Island | 3,819,765 | 524,697 | 0.29 |
| Connecticut | 11,292,876 | 1,792,584 | 0.25 |
| Michigan | 26,437,067 | 4,475,868 | 0.24 |
| Wisconsin | 16,803,467 | 2,922,637 | 0.23 |
| Minnesota | 15,264,701 | 2,869,903 | 0.21 |
| Massachusetts | 15,855,984 | 3,484,143 | 0.18 |
| California | 69,281,014 | 17,718,195 | 0.16 |
| New Hampshire | 2,384,142 | 712,035 | 0.13 |
| North Dakota | 944,189 | 390,853 | 0.10 |
| Maine | 981,830 | 659,747 | 0.06 |
| Vermont | 425,085 | 328,007 | 0.05 |
| New Mexico | 588,508 | 880,349 | 0.03 |
| Washington | 723,937 | 3,397,375 | <0.01 |
| Utah | 146,238 | 1,404,191 | <0.01 |
| Idaho | 77,081 | 752,044 | <0.01 |
| Oregon | 120,100 | 1,869,552 | <0.01 |
| Montana | 23,335 | 497,678 | <0.01 |
| Colorado | 15,688 | 2,730,333 | <0.01 |

| **Table B5.** Estimated number of worker-days exposed to occupational heat and the associated rate of exposure per 100 workers by major Standard Occupational Classification (SOC) groups across 2010-2019. | | | |
| --- | --- | --- | --- |
| **Major Occupational Groups** | **Number of worker-days exposed** | **Annual**  **average**  **worker count** | **Rate**  **per 100 workers** |
| 11 – Management Occupations | 595,408 | 15,057,059 | <0.01 |
| 13 – Business and Financial Operations Occupations | 1,339 | 7,566,490 | <0.01 |
| 15 – Computer and Mathematical Occupations | 0 | 4,330,573 | 0 |
| 17 – Architecture and Engineering Occupations | 25,934,061 | 2,810,980 | 0.37 |
| 19 – Life, Physical, and Social Science Occupations | 8,331,558 | 1,372,349 | 0.24 |
| 21 – Community and Social Service Occupations | 6,009 | 2,556,539 | <0.01 |
| 23 – Legal Occupations | 0 | 1,709,934 | 0 |
| 25 – Educational Instruction and Library Occupations | 2,171,031 | 9,093,151 | 0.01 |
| 27 – Arts, Design, Entertainment, Sports, and Media Occupations | 14,957,218 | 2,936,884 | 0.20 |
| 29 – Healthcare Practitioners and Technical Occupations | 3,367,883 | 8,737,312 | 0.02 |
| 31 – Healthcare Support Occupations | 0 | 4.181,198 | 0 |
| 33 – Protective Service Occupations | 269,695,076 | 3,252,649 | 3.32 |
| 35 – Food Preparation and Serving Related Occupations | 0 | 8,488,549 | 0 |
| 37 – Building and Grounds Cleaning and Maintenance Occupations | 583,392,007 | 5,778,925 | 4.04 |
| 39 – Personal Care and Service Occupations | 109,580,245 | 4,895,410 | 0.90 |
| 41 – Sales and Related Occupations | 62,459 | 15,684,808 | <0.01 |
| 43 – Office and Administrative Support Occupations | 144,188,846 | 18,751,535 | 0.31 |
| 45 – Farming, Fishing, and Forestry Occupations | 194,937,599 | 1,038,801 | 7.51 |
| 47 – Construction and Extraction Occupations | 1,841,924,309 | 7,544,892 | 9.77 |
| 49 – Installation, Maintenance, and Repair Occupations | 951,252,500 | 4,727,646 | 8.05 |
| 51 – Production Occupations | 543,917,356 | 8,696,996 | 2.50 |
| 53 – Transportation and Material Moving Occupations | 745,262,904 | 10,048,681 | 2.97 |

**Figure B1.** **Daily, average wet-bulb globe temperatures (WBGT, °C) in the United States.** Data used to construct occupational heat exposure estimates from 2010-2019. Yearly mean shown in red.

**Figure B2.** **Percentage of days with mean, wet bulb globe temperature ≥24°C between January 1, 2010 and December 31, 2019 by county.** Counties in grey are those with 0% of days above 24°C.

**Figure B3.** **Percentage of workers who likely work outdoors in 2019 by metropolitan/nonmetropolitan area.**

**Figure B4.** **Change in percentage of days with mean, wet bulb globe temperature ≥24°C between January 1, 2010 and December 31, 2019 by county.**

**Figure B5.** **Change in percentage of workers who likely work outdoors in 2015-2019 compared to 2010-2014 by county.**

**Figure B6. Nationwide, daily percent of outdoor workers exposed to occupational heat.** Purple line reflects screening criteria for unacclimatized workers (as shown in Figure 2a). Orange line reflects screening criteria for acclimatized workers.

**Figure B7. Trends in occupational heat exposure from 2010-2019 assuming unacclimitization.** Top panels display the average rate of occupational heat exposure per 100 workers; bottom panels display number of worker-days exposed to occupational heat. (A) Top: nationwide, annual average rate. Bottom: number of worker-days exposed by year. (B) Top: nationwide, monthly average rate. Bottom: number of worker-days exposed by month across 2010-2019. (C) Top: nationwide, annual average rate by major Standard Occupational Classification (SOC) codes. Bottom: number of worker-days exposed by major SOC codes across 2010-2019. (D) Top: annual average rate by region (South, Midwest, Northeast, and West). Bottom: number of worker-days exposed by region across 2010-2019. Note: grey, shaded areas in the top panels of (A) and (B) represent uncertainty in estimates, where the lower limit reflects the unlikely, best case scenario (where all census tracts are set at their 2.5th percentile of daily occupational heat exposure) and the upper limit reflects the unlikely, worst case scenario (where all census tracts are set at their 97.5th percentile of daily occupational heat exposure). Major Occupational Groups: 33 – Protective Service; 37 – Building and Grounds Cleaning and Maintenance; 45 – Farming, Fishing, and Forestry; 47 – Construction and Extraction; 49 – Installation, Maintenance, and Repair; 51 – Production; 53 – Transportation and Material Moving.

**Figure B8.** **Replication of Figure B7 assuming all workers are acclimatized.** Top panels display the average rate of occupational heat exposure per 100 workers; bottom panels display number of worker-days exposed to occupational heat. (A) Top: nationwide, annual average rate. Bottom: number of worker-days exposed by year. (B) Top: nationwide, monthly average rate. Bottom: number of worker-days exposed by month across 2010-2019. (C) Top: nationwide, annual average rate by major Standard Occupational Classification (SOC) codes. Bottom: number of worker-days exposed by major SOC codes across 2010-2019. (D) Top: annual average rate by region (South, Midwest, Northeast, and West). Bottom: number of worker-days exposed by region across 2010-2019. Note: grey, shaded areas in the top panels of (A) and (B) represent uncertainty in estimates, where the lower limit reflects the unlikely, best case scenario (where all census tracts are set at their 2.5th percentile of daily occupational heat exposure) and the upper limit reflects the unlikely, worst case scenario (where all census tracts are set at their 97.5th percentile of daily occupational heat exposure). Major Occupational Groups: 33 – Protective Service; 37 – Building and Grounds Cleaning and Maintenance; 45 – Farming, Fishing, and Forestry; 47 – Construction and Extraction; 49 – Installation, Maintenance, and Repair; 51 – Production; 53 – Transportation and Material Moving.

**Figure B9. Annual average rate of occupational heat exposure per 100 workers by state from 2010-2019 assuming all workers are unacclimatized**. The dotted red line reflects the national average rate across 2010-2019.
